# Determination of Total Polysaccharides and Total Flavonoids in *Chrysanthemum morifolium* Using Near-Infrared Hyperspectral Imaging and Multivariate Analysis

**DOI:** 10.3390/molecules23092395

**Published:** 2018-09-19

**Authors:** Juan He, Lidan Chen, Bingquan Chu, Chu Zhang

**Affiliations:** 1 Key Laboratory of Research and Development of Chinese Medicine of Zhejiang Province, Zhejiang Academy of Traditional Chinese Medicine, Hangzhou 310007, China; hej0516@126.com; 2 College of Automobile Technology, Zhejiang Technical Institute of Economics, Hangzhou 310018, China; cld1121@126.com; 3 School of Biological and Chemical Engineering, Zhejiang University of Science and Technology, Hangzhou 310023, China; 4 College of Biosystems Engineering and Food Science, Zhejiang University, Hangzhou 310058, China; 5 Key Laboratory of Spectroscopy Sensing, Ministry of Agriculture, Hangzhou 310058, China

**Keywords:** near-infrared hyperspectral imaging, *Chrysanthemum morifolium*, total polysaccharides, total flavonoids

## Abstract

The rapid and nondestructive determination of active compositions in *Chrysanthemum morifolium* (Hangbaiju) is of great value for producers and consumers. Hyperspectral imaging as a rapid and nondestructive technique was used to determine total polysaccharides and total flavonoids content in *Chrysanthemum morifolium*. Hyperspectral images of different sizes of *Chrysanthemum morifolium* flowers were acquired. Pixel-wise spectra within all samples were preprocessed by wavelet transform (WT) followed by standard normal variate (SNV). Partial least squares (PLS) and least squares-support vector machine (LS-SVM) were used to build prediction models using sample average spectra calculated by preprocessed pixel-wise spectra. The LS-SVM model performed better than the PLS models, with the determination of the coefficient of calibration (R^2^c) and prediction (R^2^p) being over 0.90 and the residual predictive deviation (RPD) being over 3 for total polysaccharides and total flavonoids content prediction. Prediction maps of total polysaccharides and total flavonoids content in *Chrysanthemum morifolium* flowers were successfully obtained by LS-SVM models, which exhibited the best performances. The overall results showed that hyperspectral imaging was a promising technique for the rapid and accurate determination of active ingredients in *Chrysanthemum morifolium*, indicating the great potential to develop an online system for the quality determination of *Chrysanthemum morifolium*.

## 1. Introduction

Tea is one of the most popular beverages throughout the world. Flowering tea is an important kind of tea; it contains the flowers and wrapped leaves of the plant. Flowering tea harvested from *Chrysanthemum morifolium* (Hangbaiju) is a famous kind of tea in China, due to its unique taste and aroma. *Chrysanthemum morifolium* are planted in several regions in China; however, *Chrysanthemum morifolium* planted in Tongxiang, Zhejiang Province, China are the most famous [1]. Besides the taste and aroma, medical benefits are the other contributors to the fame of *Chrysanthemum morifolium*. These benefits include reducing eye strain, an antipyretic effect, a sedative effect, and reducing blood pressure [2].

Polysaccharides and flavonoids are major active ingredients in *Chrysanthemum morifolium*, and contribute greatly to its medical value. The quantitative measurement of concentrations of polysaccharides and flavonoids in *Chrysanthemum morifolium* is important for quality sorting and consumption. The spectrophotometric method is used in China as standard reference measurement method to determine polysaccharides and flavonoids content in *Chrysanthemum morifolium* [3]. Methods such as HPLC [4] and gas chromatography-mass spectrometry (GC-MS) [5] have also been studied for active ingredients measurement. These methods can accurately measure the concentrations of polysaccharides, flavonoids, and some other active ingredients. However, these methods are time-consuming, reagent-dependent, expensive, and require complex operation skills. More importantly, these methods usually measure a small number of samples, and thus only a small set of samples can be measured in order to represent a larger amount. These methods are not suitable for large-scale measurement.

On the other hand, the flowering tea of *Chrysanthemum morifolium* is harvested according to its blooming stage. The unopened flower buds are harvested, known as the famous ‘Taiju’, fetching a high price in the marketplace. The blooming flowers are also harvested and sold at a lower price. *Chrysanthemum morifolium* is manually harvested by experienced farmers. During the harvest, it is quite difficult to determine which flower should be harvested. The quality grading is simple, and the quality grading approach can be improved to achieve better products with greater economic value. Rapid and nondestructive quality grading of *Chrysanthemum morifolium* according to their taste, aroma, and active ingredients is needed due to the development of modern measurement techniques and instruments.

To overcome these issues, rapid and nondestructive techniques are needed. In recent years, hyperspectral imaging, integrating the spectroscopy technique and imaging technique, has been employed to provide spatial and spectral information simultaneously. Hyperspectral imaging has been used to detect various compositions of samples as a rapid and nondestructive technique, such as herbal medicine [6,7], food [8,9,10,11], and agriculture [12,13]. Beyond building detection models, hyperspectral imaging has also been used to obtain prediction visualization images to explore the composition distribution within and among samples [14,15,16].

The objective of this study was to investigate the feasibility of using near-infrared hyperspectral imaging to determine total polysaccharides and total flavonoids in *Chrysanthemum morifolium*. The specific objectives were (1) to develop quantitative detection models by partial least squares (PLS) and least squares-support vector machine (LS-SVM), (2) to evaluate the performance of spectral preprocessing, and (3) to form prediction visualization maps of the total polysaccharides and total flavonoids content.

## 2. Results and Discussion

### 2.1. Spectral Profiles and Spectral Preprocessing

**Figure 1:**
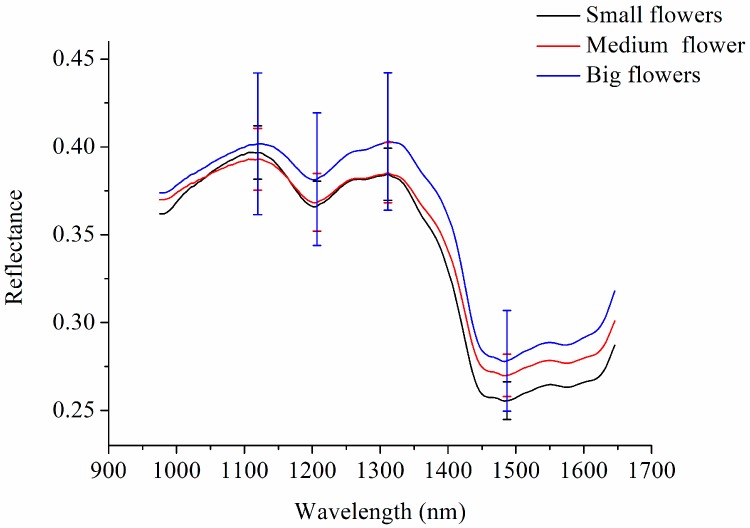
Average spectra with standard deviation (SD) at typical wavelengths of small flowers, medium flowers, and big flowers.

Spectra data were extracted from the hyperspectral images of the three different kinds of *Chrysanthemum morifolium*, including small flower (the degree of openness of the tubular flower is less than 10%, namely ‘Taiju’), medium flower (the degree of openness of the tubular flower is 30–50%), and big flower (the degree of openness of the tubular flower is 70–90%). Because pixel-wise spectra in the hyperspectral images contained random noises, wavelet transform (WT) using wavelet function Daubechies 8 and decomposition level 3 was first applied to smooth the pixel-wise spectra [17]. The average pixel-wise spectra preprocessed by WT are presented in Figure 1. In addition, the standard deviation (SD) of four typical spectral bands (peaks around 1119 nm and 1311 nm, and valleys around 1210 nm and 1487 nm) are presented.

The spectral peaks around 1119 and 1311 nm and the spectral valley around 1210 nm might be attributed to the second overtone of C–H stretch [18]. The spectral valley around 1487 nm may be attributed to the first overtone of O–H stretch [19].

The average reflectance spectrum of big flowers was obviously higher than the average spectra of medium flowers and small flowers; the reason for this might be the difference in size. The size of big flowers is larger than that of medium and small flowers, and the distance between the detector and the sample of big flowers is closer, resulting in a higher reflectance value. As seen from the standard deviation, larger variations could be observed for big flowers, and overlaps could also be observed among the three kinds of samples.

Standard normal variate (SNV) is a widely used spectral preprocessing method to reduce the influence of particle size, optical path variation, and surface scatter on spectral data. Thus, pixel-wise spectra preprocessed by WT were also preprocessed by SNV [20].

### 2.2. Statistical Analysis and Sample Set Split

**Figure 2:**
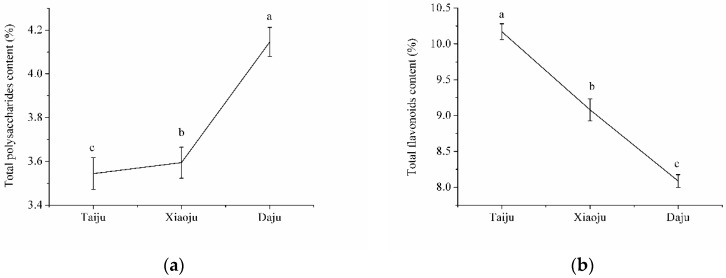
Statistical analysis of total polysaccharides content and total flavonoids content in *Chrysanthemum morifolium*: (**a**) total polysaccharides; (**b**) total flavonoids. The letters a, b, c in the figures indicate the results of significant analysis of small flowers, medium flowers, and big flowers.

The statistical analysis of the measured total polysaccharides and total flavonoids content of small flowers, medium flowers, and big flowers is shown in Figure 2. The total polysaccharides content increased in the order of small flowers, medium flowers, and big flowers, while the total flavonoids content decreased in the order of small flowers, medium flowers, and big flowers. The differences of total polysaccharides and total flavonoids content in small flowers, medium flowers, and big flowers were significant, indicating that it was meaningful to harvest *Chrysanthemum morifolium* according to different blooming stages.

To quantitatively determine the total polysaccharides and total flavonoids content, the samples were split into the calibration and prediction sets for model establishment. In total, 279 samples were collected, and the samples were ranked from low to high based on the total polysaccharides and total flavonoids content, respectively. For every three samples, the first sample and the third sample were selected for the calibration set, and the remaining samples were selected for the prediction set. There were 186 samples in the calibration set and 93 samples in the prediction set for total polysaccharides and total flavonoids content. The statistical summary of the calibration set and the prediction set are presented in Table 1.

### 2.3. Model Development

To quantitatively detect total polysaccharides and total flavonoids content, PLS and LS-SVM models were developed using the spectra averaged from pixel-wise spectra preprocessed by WT and the spectra averaged from pixel-wise spectra preprocessed by WT and SNV. The results are shown in Table 2 and Table 3. For clarity, the spectra averaged from pixel-wise spectra preprocessed by WT are referred to as WT preprocessed spectra, and the spectra averaged from pixel-wise spectra preprocessed by WT and SNV are termed WT-SNV preprocessed spectra.

For total polysaccharides content detection, the results are shown in Table 2. The PLS model using WT preprocessed spectra exhibited a good performance, with a coefficient of calibration (R^2^c) of 0.89, a coefficient of prediction (R^2^p) of 0.90, and a residual predictive deviation (RPD) over 3. The LS-SVM model using WT preprocessed spectra obtained similar results. The PLS model using WT-SNV preprocessed spectra obtained relatively worse results, with an RPD under 2.5, while the LS-SVM model using WT-SNV preprocessed spectra obtained the best results, with an R^2^c of 0.94, R^2^p of 0.93, and RPD of 3.73. The performances of the PLS models exhibited larger variations than those of the LS-SVM models due to the different spectral preprocessing methods.

For total flavonoids content detection, the results are shown in Table 3. Better results of calibration models were observed compared with those employed for the polysaccharides content detection. The PLS model using WT preprocessed spectra obtained good results with an R^2^c of 0.95, R^2^p of 0.96, and RPD of 4.78, indicating excellent prediction performance. The PLS model using WT-SNV preprocessed spectra obtained worse results, with an R^2^c of 0.96, R^2^p of 0.87, and RPD of 1.76. The LS-SVM model using WT preprocessed spectra achieved an excellent performance, with an R^2^c of 0.97, R^2^p of 0.98, and RPD of 6.62. Meanwhile, the LS-SVM model using WT-SNV preprocessed spectra also exhibited an excellent performance, with an R^2^c of 0.97, R^2^p of 0.94, and RPD of 4.10. Similarly, the performances of the PLS models exhibited larger variations than those of the LS-SVM models due to the different spectral preprocessing methods.

As seen from Table 2 and Table 3, the PLS models showed larger variations under different spectral preprocessing methods. The LS-SVM models outperformed the PLS models. This might be because there was nonlinear information in the spectra. The results of the LS-SVM models for total polysaccharides content and total flavonoids content detection indicated an excellent prediction performance. The overall results showed that hyperspectral imaging was a promising technique for the rapid, nondestructive, and accurate determination of total polysaccharides content and total flavonoids content in *Chrysanthemum morifolium*.

The detection of polysaccharides content in biological samples using near-infrared spectral information has been conducted thanks to the rapid and nondestructive detection abilities of near-infrared spectroscopy. Ma et al. (2018) used this method to detect the polysaccharides in Mycelia of the genus Ganoderma, with an R^2^p over 0.9 and RPD over 3 [21]. Zhang et al. (2015) used near-infrared spectroscopy to detect the polysaccharides in Glycyrrhiza, with an R^2^p over 0.9 [22]. Chen et al. (2012) used near-infrared spectroscopy to detect the polysaccharides in *Ganoderma lucidum* and *Ganoderma atrum*, with an R^2^p over 0.9 [23]. Wang et al. (2018) used near-infrared spectroscopy to determine the polysaccharides in *Radix codonopsis*, with a correlation coefficient of prediction (Rp) over 0.9 [24]. These results have indicated the feasibility of using spectral information in the near-infrared region to detect polysaccharides.

Some studies have also used near-infrared spectral information to detect flavonoids content in biological samples. Cai et al. (2012) used near-infrared spectroscopy to detect the content of various flavonoids in propolis, and the prediction of total flavonoids content had an R^2^p over 0.8 and RPD over 3 [25]. Shi et al. (2012a) used near-infrared spectroscopy to detect total flavonoids content in fresh *Ginkgo biloba* leaf, and the optimal model achieved an R^2^p over 0.8 [26]. Shi et al. (2012b) again used near-infrared spectroscopy to detect flavonoids content in fresh *Ginkgo biloba* leaf, and the optimal mode achieved an R^2^p over 0.8 [27]. Shen et al. used near-infrared spectroscopy to detect flavonoids content in Goji berry, realizing an Rp over 0.9 in the optimal model [28]. These results, along with the results of this study, indicate the feasibility of using spectral information in the near-infrared region to detect flavonoids content in biological samples.

### 2.4. Prediction Maps

The ability to form prediction maps is one of the major advantages of hyperspectral imaging; it is the typical application of hyperspectral imaging. As shown in Table 2 and Table 3, the LS-SVM model using WT-SNV preprocessed spectra performed best for total polysaccharides content determination, and the LS-SVM model using WT preprocessed spectra performed best for total flavonoids content determination. These two models were used to form prediction maps for total polysaccharides content and total flavonoids content in *Chrysanthemum morifolium*. One common hyperspectral image of Taiju, one common hyperspectral image of Xiaoju, and one common hyperspectral image of Daju in the prediction sets of the two sample sets for both total polysaccharides content and total flavonoids content were used to form the prediction maps.

**Figure 3:**
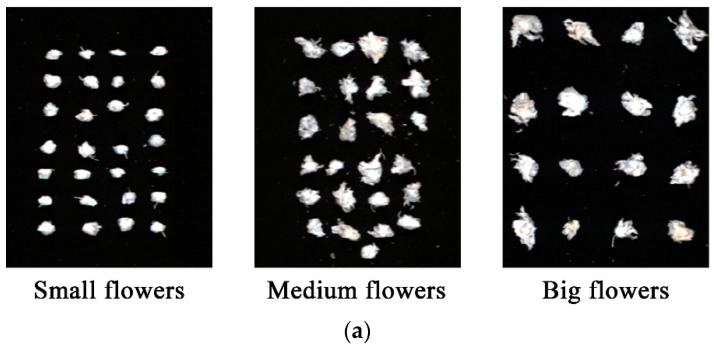

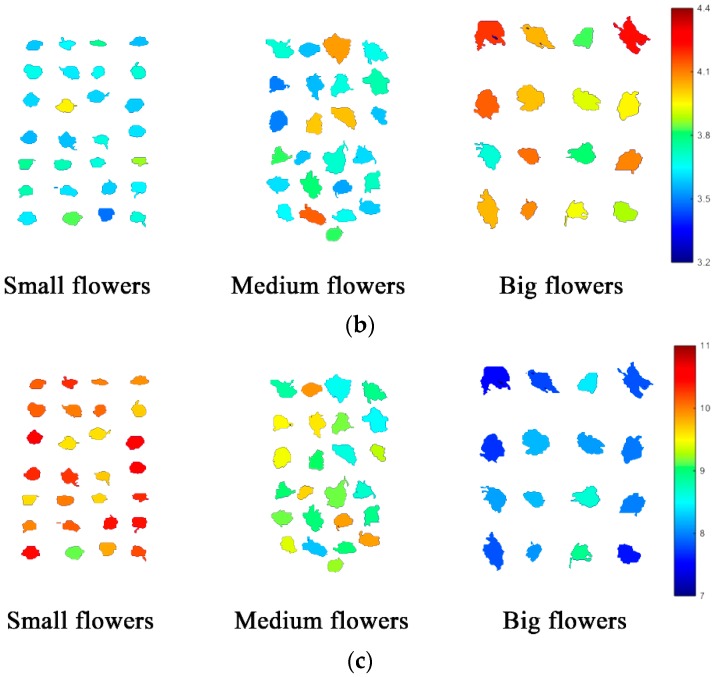
Prediction maps of total polysaccharides content and total flavonoids content in *Chrysanthemum morifolium*: (**a**) pseudocolor images; (**b**) prediction maps of total polysaccharides content; (**c**) prediction maps of total flavonoids content.

The general procedure of image visualization first involves the preprocessing of the pixel-wise spectra. Then, the average spectrum of each flower was calculated, and the total polysaccharides content or total flavonoids content was predicted using the corresponding LS-SVM model. The prediction value was then assigned to each pixel within the flower. The image with each pixel of the total polysaccharides content or total flavonoids content was then formed, and a colorbar was used to represent the content by different colors. The prediction maps of total polysaccharides content and total flavonoids content in small flowers, medium flowers, and big flowers are shown in Figure 3. It was a fact that quality sorting of *Chrysanthemum morifolium* was mainly based on each single flower. Thus the average prediction value of each flower was visualized, and the differences among different flowers were explored.

Figure 3a shows the pseudocolor images extracted from the hyperspectral images of small flowers, medium flowers, and big flowers (formed by grayscale images at 1000 nm, 1200 nm, and 1400 nm). It was observed that the sizes of the flowers were different. The corresponding prediction maps of total polysaccharides content are presented in Figure 3b. The prediction maps showed the trend that the total polysaccharides content was ranked from high to low in the order of big flowers, medium flowers, and small flowers. The prediction maps of total flavonoids content are shown in Figure 3c. The prediction maps showed the trend that the total flavonoids content was ranked from low to high in the order of big flowers, medium flowers, and small flowers. The trends of the total polysaccharides content and total flavonoids content in the prediction maps were consistent with the trends shown in Figure 2. The results indicated that the prediction maps developed from hyperspectral imaging can be used to present the distribution of chemical compositions among different samples. Using prediction maps, the quality of *Chrysanthemum morifolium* can be visualized and graded, indicating the potential of developing online rapid and nondestructive detection systems.

## 3. Materials and Methods

### 3.1. Sample Preparation

The samples of *Chrysanthemum morifolium* were harvested from a plantation in Tongxiang, Zhejiang Province, China. According to the degree of openness of the tubular flower, the flowers were collected into three different kinds, including small flowers (the degree of openness of the tubular flower is less than 10%), medium flowers (the degree of openness of the tubular flower is 30–50%), and large flowers (the degree of openness of the tubular flower is 70–90%). Fresh *Chrysanthemum morifolium* was harvested from different plants in the plantation by experienced planters, and packed together as small flowers, medium flowers, and large flowers. Enzymes in the flowers were deactivated via steam treatment for 90 s, and the flowers were subsequently dried at 60 °C until constant weight. For each kind of dry flower, 3 g of flowers were randomly collected as a sample, and 93 samples of each kind of flower were collected.

### 3.2. Hyperspectral Image Acquisition

#### 3.2.1. Hyperspectral Imaging System

A near-infrared hyperspectral imaging system covering the spectral range of 874 to 1734 nm (256 bands) was used for the image acquisition of dry *Chrysanthemum morifolium*. The hyperspectral imaging system contains the following components.

(1) Imaging module: An imaging spectrograph (ImSpector N17E; Spectral Imaging Ltd., Oulu, Finland) coupled with a high-performance camera (Xeva 992; Xenics Infrared Solutions, Leuven, Belgium) and a camera lens (OLES22; Specim, Spectral Imaging Ltd., Oulu, Finland). The pixel size was 30 μm × 30 μm. The spatial dimension of the acquired image was 326 × y × 256, where the number 326 is the width of the image, y is the length of the image which was determined manually, and the number 256 is the number of bands.
(2) Illumination module: A line light source system containing a 150 W tungsten halogen lamp (3900e Lightsource; Illumination Technologies Inc.; West Elbridge, NY, USA). Lights were transferred into two optical fibers. The two fibers were symmetrically placed on either side of the imaging module.
(3) Sample movement module: A moving plate driven by a stepper motor (Isuzu Optics Corp., Taiwan, China). The path length of the stepper motor was 400 mm.
(4) Software: A data acquisition and preprocessing software (Xenics N17E, Isuzu Optics Corp., Taiwan, China). The settings of the image acquisition parameters and the moving speed of the moving plate were controlled using the software.

#### 3.2.2. Image Acquisition and Calibration

**Figure 4:**
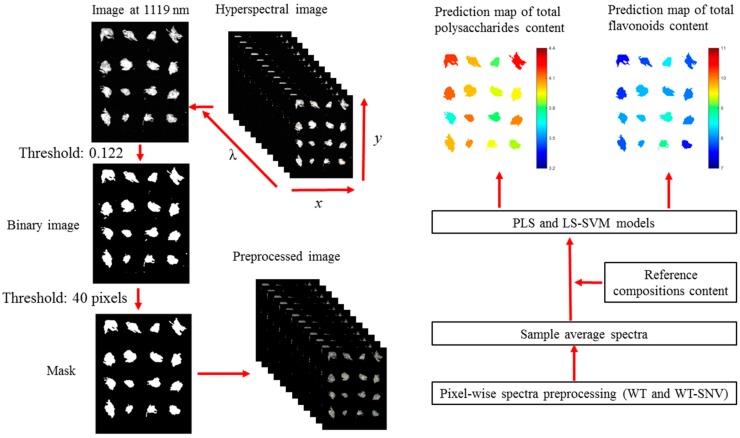
Flowchart of data analysis procedures.

To acquire clear and nondeformed images, the system parameters for image acquisition were optimized after trials as follows: camera exposure time of 3000 us; distance between the camera lens and the moving plate of 16 cm; and moving speed of the plate of 14.3 mm/s. For image acquisition, single flowers were placed separately (as shown in Figure 4).

The acquired hyperspectral images were raw images. To reduce the influence of dark current and changes in light intensity at different wavelengths, the raw images should be calibrated. The image calibration was conducted based on the follow equation,

(1) $$I_{R}=\frac{I_{raw}-I_{dark}}{I_{white}-I_{dark}}$$

where $I_{R}$ is the calibrated image, $I_{raw}$ is the raw image, $I_{white}$ is the white reference image, and $I_{dark}$ is the dark reference image. The dark reference image was acquired by turning off the light source together with covering the camera lens completely with its opaque cap. The dark reference image was to remove the influence of dark current on the camera. The white reference image was acquired by using a white Teflon tile with nearly 100% reflectance for light intensity calibration.

### 3.3. Spectral Data Extraction

After image calibration, the spectral information of dry *Chrysanthemum morifolium* was extracted from the hyperspectral images. The whole spectral data extraction procedure was conducted on Matlab R 2014b (The Math Works, Natick, MA, USA). The general procedure of spectral data extraction can be summarized as follows.

(1) Background removal: The essential procedure for spectral data extraction involved removing the background information. The reflectance between the sample region and background region is different, and the grayscale image at the 74th waveband (1119 nm)—which showed the largest difference between the sample region and background region—was selected to build a binary image. The reflectance threshold value was set as 0.122. Then, the isolated small objects caused by noise in the image were removed by setting an area threshold value of 40 pixels.
(2) Spectral data extraction: According to a previous paper [17], the pixel-wise spectra contained obvious noises. After background removal, pixel-wise spectra were then preprocessed by WT using discrete wavelet function Daubechies 8 and decomposition level 3 to smooth the spectra. The pixel-wise spectra preprocessed by WT were treated in two different ways. Firstly, the pixel-wise spectra within the sample region were averaged as the sample spectrum. Secondly, the pixel-wise spectra were preprocessed by SNV, and the preprocessed spectra within the sample region were averaged as the sample spectrum.

### 3.4. Reference Measurement of Total Polysaccharides and Total Flavonoids

The total polysaccharides and total flavonoids content in *Chrysanthemum morifolium* were measured by the methods introduced in the Chinese Pharmacopoeia [3]. Details of the measurement of total polysaccharides and total flavonoids content are described below.

#### 3.4.1. Total Polysaccharides Content Measurement

To measure the total polysaccharides content, a standard curve is first developed. A reference glucose solution with a concentration of 103 μg/mL was prepared using standard anhydrous glucose and distilled water. Then 0.2 mL, 0.4 mL, 0.6 mL, 0.8 mL, and 1.0 mL of reference solution were accurately measured and placed into a 10-mL test tube with a glass stopper, and water was added into each tube to set the solution volume to 1 mL. After volume calibration, 1 mL of 5% phenol solution was added and the tubes were shaken thoroughly; then, 5 mL sulfuric acid was added and the tubes were shaken thoroughly. The tubes were heated in a boiling water bath for 20 min, and then cooled in an ice bath for 5 min. The corresponding reagent in a tube was used as blank. Finally, the absorbance at 488 nm of each solution was measured by an ultraviolet-visible spectrophotometer (TU-1810S, Beijing Purkinje General Instrument Co., Ltd. Beijing, China), and the standard curve was obtained. The standard curve in this study was A = 9.9175C + 0.0367 (R^2^ = 0.9995), where A is the absorbance and C is the concentration.

After standard curve development, a 0.25-g sample of the grounded flower powders (filtered through a sieve with a bore diameter of 2.0 mm) was placed into a round-bottom flask, to which was added 50 mL 80% ethanol. The flask was placed in a hot water bath (80 °C) for 1 h. Then, the solution was filtered and the residuals were washed three times with 10 mL hot 80% ethanol. The residuals were then placed in the flask, and 60 mL distilled water was added. The flask was placed in a hot water bath (80 °C) for 1 h. The same procedures of filtration and washing were conducted. Then, the resultant solutions were gathered, cooled, and transferred into a 100-mL volumetric flask; the solution volume was set to 100 mL by adding distilled water. After the sample solution preparation, 0.5 mL solution was added to a 10-mL test tube with a glass stopper. Subsequently, the same process as that employed for the development of the standard curve was followed. The total polysaccharides content were calculated using the measured absorbance and the standard curve. For each sample, three replications were conducted, and the average value was used as a reference total for the polysaccharide content of the sample. The average recovery of total polysaccharides using this method was 96.7%, and relative standard deviation (RSD) was 2.89%.

#### 3.4.2. Total Flavonoids Content Measurement

To measure the total flavonoids content, a standard curve was first developed. A reference rutin solution with a concentration of 0.2491 mg/mL was prepared using standard anhydrous rutin and distilled water. Then 1 mL, 2 mL, 3 mL, 4 mL, 5 mL, and 6 mL reference solution were placed into 25-mL volumetric flasks, and the solution volume was set to 6 mL by adding distilled water. After volume calibration, 1 mL 5% sodium nitrite solution was added and the mixture was shaken thoroughly; then, 1 mL 10% aluminum nitrate solution was added and the mixture was shaken thoroughly. After standing for 6 min, 10 mL sodium hydroxide solution was added and the solution volume was set to 25 mL by adding distilled water. The solution was shaken thoroughly and then left to stand for 15 min. The corresponding reagent in a flask was used as blank. Then the absorbance at 500 nm of each solution was measured by an ultraviolet-visible spectrophotometer, and the standard curve was obtained. The standard curve in this study was A = 0.0134C − 0.0243 (R^2^ = 0.9996), where A is the absorbance and C is the concentration.

After the standard curve development, a 0.25-g sample of the ground flower powders (filtered through a sieve with a bore diameter of 2.0 mm) was placed into a conical flask with a glass stopper. Then, 50 mL 75% ethanol was added and the flask was sealed and weighed. The solution was processed with ultrasonic vibration for 30 min at a temperature of 60 °C. The flask was cooled and weighed; the weight loss was compensated by adding 75% ethanol. The solution was shaken thoroughly and filtered, after which 2 mL of filtered solution was collected and placed into a 25-mL volumetric flask. Subsequently, the same process as that employed for the development of the standard curve was followed. The total flavonoids content was calculated using the measured absorbance and the standard curve. For each sample, three replications were conducted, and the average value was used as reference for total flavonoids content of the sample. The average recovery of total flavonoids using this method was 97.5%, and RSD was 2.35%.

### 3.5. Multivariate Data Analysis Methods

Data analysis is essential for the interpretation of hyperspectral images. The general data analysis procedure in this study included spectral preprocessing and calibration model establishment.

Wavelet transform (WT) was primarily used to reduce the obvious random noises. However, the spectral information can be also affected by scattering effects and background information. Due to the influence of particle size, optical path variation, and surface scatter, standard normal variate (SNV) was also explored to preprocess the pixel-wise spectra after WT preprocessing.

The preprocessed spectra were then inputted into partial least squares (PLS) models [29] and least squares-support vector machine (LS-SVM) models [30] to evaluate the influence of preprocessing methods. PLS is a linear regression method which explores the linear relationship between X (spectra) and Y (chemical attributes). PLS decomposes X and Y into new data spaces at the same time, and tries to maximize the covariance between the new data components of X and Y. The number of latent variables (LVs) is determined for optimal PLS models.

LS-SVM is an extension of support vector machine (SVM). LS-SVM uses a set of linear equations as constraints instead of a convex quadratic programming (QP) for SVM. LS-SVM has the advantages of fast computation and good generalization ability. LS-SVM is a kernel-based machine learning method, and the selection of kernel function is important. Radial basis function (RBF) is the widely used kernel function in spectral data analysis, and RBF was used in this study. The model parameter γ and the kernel parameter σ^2^ should be determined, and a grid-search procedure is used to find the optimal combination of the two parameters.

To obtain optimal models, leave-one-out cross-validation procedure is applied for the PLS and LS-SVM models. The model performances were evaluated according to the determination of the coefficient (R^2^), root mean square error (RMSE) of the calibration, prediction, and cross-validation. In addition, the residual predictive deviation (RPD) was also used to evaluate the model performances. According to a past paper [31], an R^2^ between 0.61 and 0.8 indicates that the models could be used for prediction. An R^2^ between 0.81 and 0.9 indicates that the models performed well, and an R^2^ over 0.9 indicates excellent prediction performances. An RPD under 2 indicates that models could not be used for prediction, while an RPD between 2 and 2.5 indicates that the models could be used for prediction, and an RPD between 2.5 and 3 indicates that the models performed well. Finally, an RPD over 3 indicates excellent performances of the models.

### 3.6. Development of Prediction Maps

A well-known advantage of hyperspectral imaging is the ability to form prediction visualization maps. Based on models established by spectral information, the similar pixel-wise spectral information can be extracted and predicted. Then the physical and chemical properties of each pixel can be predicted. However, it should be noticed that it was useless to predict the pixel-wise polysaccharide and flavonoid content of each individual *Chrysanthemum morifolium* for quality sorting. The total polysaccharides and total flavonoids content of each individual *Chrysanthemum morifolium* would be better used for quality grading and sorting. Thus, the total polysaccharides and total flavonoids of each individual flower were predicted and visualized instead of the prediction of each pixel. The procedure employed to form the prediction maps is similar to that reported in a previous papaer [32]. The flowchart of the whole image processing and visualization procedure of this study is presented in Figure 4.

## 4. Conclusions

Hyperspectral imaging was used to determine total polysaccharides and total flavonoids contents of *Chrysanthemum morifolium*. Hyperspectral images in the spectral range of 975 to 1646 nm (200 bands) were used for analysis. Spectral profiles indicated that the particle size of flowers affected the spectral features. Pixel-wise spectra preprocessed by WT and those preprocessed by WT with SNV were explored. PLS models and LS-SVM models were built using the preprocessed spectra. While all models obtained acceptable results, the LS-SVM model using WT-SNV preprocessed spectra obtained the best performance for total polysaccharides content determination, with an R^2^c of 0.94, R^2^p of 0.93, and RPD of 3.73; the LS-SVM model using WT preprocessed spectra obtained the best performance for total flavonoids content determination, with an R^2^c of 0.97, R^2^p of 0.98, and RPD of 6.62. Both prediction models indicated that hyperspectral imaging was a promising technology to determine total polysaccharides and total flavonoids in *Chrysanthemum morifolium*. The prediction maps of total polysaccharides and total flavonoids in *Chrysanthemum morifolium* indicated that it was feasible to obtain online visualization images for online quality detection and sorting of different *Chrysanthemum morifolium* flowers. Compared with the reference methods for total polysaccharides and total flavonoids measurement using ultraviolet-visible spectrometry, hyperspectral imaging was more environmentally friendly and more efficient in handling large amounts of samples, and predictions could be made for each single flower. The results in this study may help to develop rapid and nondestructive online quality detection and sorting systems for *Chrysanthemum morifolium* using hyperspectral imaging.

## Figures and Tables

**Table 1:** Statistical summary of samples in the calibration set and the prediction set.

	Calibration			Prediction		
	Range (%)	Mean (%)	SD (%)	Range	Mean (%)	SD (%)
Total polysaccharides	3.37–4.35	3.76	0.28	3.40–4.34	3.76	0.28
Total flavonoids	7.81–10.43	9.11	0.86	7.84–10.42	9.11	0.86

**Table 2:** Prediction results of total polysaccharides content in *Chrysanthemum morifolium*.

			Calibration Set				Prediction Set		
		Parameters ^a^	R^2^c ^b^	RMSEC	R^2^cv	RMSECV	R^2^p	RMSEP	RPD
PLS	WT	5	0.89	0.095	0.87	0.10	0.90	0.089	3.15
	WT-SNV	1	0.81	0.12	0.81	0.12	0.83	0.12	2.33
LS-SVM	WT	5.1072 × 10^5^, 1.1169 × 10^4^	0.94	0.070	0.90	0.087	0.90	0.091	3.08
	WT-SNV	5.7740, 182.3955	0.94	0.070	0.90	0.088	0.93	0.075	3.73

^a^: Parameters of the partial least squares (PLS) model and least squares-support vector machine (LS-SVM) model. For the PLS model, the model parameter is the optimal number of latent variables (LVs); for LS-SVM, the model parameters are the regularization parameter γ and the kernel parameter σ^2^. R^2^c ^b^: coefficient of determination of calibration; R^2^p: coefficient of determination of prediction; R^2^cv: coefficient of determination of cross-validation; WT: wavelet transform; SNV: standard normal variate; RMSEC: root mean square error of calibration; RMSECV: root mean square error of cross-validation; RMSEP: root mean square error of prediction, RPD: residual predictive deviation.

**Table 3:** Prediction results of total flavonoids content in *Chrysanthemum morifolium*.

			Calibration Set				Prediction Set		
		Parameters	R^2^c	RMSEC	R^2^cv	RMSECV	R^2^p	RMSEP	RPD
PLS	WT	4	0.95	0.20	0.94	0.20	0.96	0.18	4.78
	WT-SNV	3	0.96	0.18	0.95	0.19	0.87	0.49	1.76
LS-SVM	WT	6.7832 × 10^5^, 1.4727 × 10^4^	0.97	0.14	0.96	0.18	0.98	0.13	6.62
	WT-SNV	13.9882, 716.0323	0.97	0.14	0.97	0.16	0.94	0.21	4.10
